# Minority status, stigma, prejudice and bullying: growing up in jeopardy

**DOI:** 10.1192/bji.2021.29

**Published:** 2021-08

**Authors:** David Skuse

**Affiliations:** Professor of Behavioural and Brain Sciences, Division of Population, Policy and Practice, UCL Great Ormond Street Institute of Child Health, London, UK. Email: d.skuse@ucl.ac.uk

**Keywords:** Roma, intellectual disability, refugees, bullying, discrimination

## Abstract

Stigma and alienation are suffered by many individuals with mental health disorders, in societies around the world. Rejection is all the more common among those who have intellectual disabilities or who are from ethnic minorities. In this issue, three papers consider the suffering experienced by patients with vulnerabilities that militate against their being in receipt of the psychiatric care they assuredly deserve.

In this issue of the journal, we are considering problems faced by our peers who do not have our advantages in terms of education or opportunities to integrate into society, either because of intellectual disability or because of their minority status.

## The triple jeopardy of intellectual disability

In a report from Roy and colleagues, we learn about the World Psychiatric Association's Presidential Action Plan (2020–2023), which provides guidance on the future integration of medical, social and a rights-based models of care.^1^ The article concerns intellectual disabilities, an important component of this initiative, which focuses on the plight of people living in low- and middle-income countries. Prejudice and stigma lead to their needs being ignored and their quality of life is at risk of deteriorating, with a lack of opportunity to participate fully in society. The impact of discrimination is greatest on children with intellectual disabilities, and the authors highlight the triple jeopardy of disability, poverty and gender, for females are particularly likely to suffer disadvantage. A call to action emphasises the potential value of community care.

## Mental healthcare of the Greece's Roma population

In many ways, the issues raised by Roy et al are echoed in the second article on our theme, which concerns the Roma population who live in the far Eastern regions of Greece, where they represent about 1 in 12 of the population.^2^ As a group, the Roma have traditions that are quite distinct from those of the majority, with a different language (Turkish) and a different religion (Islam). Lack of integration and hence education has led to high rates of illiteracy and, consequently, unemployment. Gender inequality has a major impact on the lives of Roma girls, who are usually married by 17 years of age. Roma people who present with mental health problems such as depression and anxiety are proportionally more often female. They face challenges in receiving appropriate treatment. Barriers to care are associated with a high drop-out rate, which could be alleviated in part by providing more community-based services.

## Bullying of refugee children in Greek schools

The Roma are long-standing immigrants in Greece, but more recently that country has faced a wave of immigration from the Middle East, as a consequence of the conflicts that persist in that region, coming especially from Syria and Afghanistan. Alexandra Gkouliama and her colleagues discuss the problems that refugee children (who were often unaccompanied) face in Greek schools, where their ethnicity puts them at high risk of being the victims of bullying.^3^ Until 30 years or so ago, Greece was ethnically a relatively homogeneous country, and the rapid adaptation demanded of society to this refugee influx has inevitably introduced tensions. Bullying has serious consequences for mental health wherever and whenever it occurs, and the sequelae of childhood bullying persist into adulthood. The integration of refugee children into Greek society will be compromised by these adverse experiences, but fortunately efforts are being made to manage the situation through political initiatives.
